# Maintaining international research collaborations in the setting of a pandemic: Approach in Indonesia

**DOI:** 10.7189/jogh.11.03087

**Published:** 2021-07-17

**Authors:** Muhammad Karyana, Herman Kosasih, Aaron T Neal, Chuen-Yen Lau

**Affiliations:** 1 Center for Human Resources and Health Services Research and Development, National Institute of Health Research and Development, Ministry of Health, Jakarta, Indonesia; 2 Indonesia Research Partnership on Infectious Disease (INA-RESPOND), Jakarta, Indonesia; 3 Collaborative Clinical Research Branch, Division of Clinical Research, National Institute of Allergy and Infectious Diseases, National Institutes of Health, Bethesda, Maryland, USA; 4 Host-Virus Interaction Branch, Center for Cancer Research, National Cancer Institute, National Institutes of Health, Bethesda, Maryland, USA

## COVID-19 EMERGENCE AND IMPACT

The emergence and global spread of SARS-CoV-2 is the latest and most significant pandemic in recent history. Despite its extraordinary impact, an outbreak of this scale is not entirely unexpected given the pattern of animal-to-human spillover events that has accelerated over time. H1N1 influenza in 1918 and 2009, SARS-CoV in 2003, MERS-CoV in 2012, and Ebola in West Africa in 2014 are just a few significant outbreaks from previously known or unknown pathogens that have occurred and will continue to occur as human-animal interactions and ecological disruption increase [1]. While previous epidemics have been limited by specific and targeted public health interventions, SARS-CoV-2 has necessitated an unprecedented international response, including city-wide lockdowns, travel restrictions, mass quarantines, social distancing measures, and work from home policies.

The enormous impact of COVID-19 requires a global approach to combat its sequelae, including shifting research priorities to address disease epidemiology, pathogenesis, clinical course, diagnostic approaches, and prevention strategies. To support these research needs, and to protect study volunteers and staff from SARS-CoV-2 exposure, many non-COVID-19 activities have been paused to allow redirection of critical resources and expertise. However, rapid research implementation during a pandemic situation can be hindered by a lack of capacity. Establishment and maintenance of collaborative research networks in times of calm reduces many of the obstacles through pre-existing physical, human, administrative, and intellectual capacity. For example, the collaboration may already have a reference laboratory, established sites and investigators, centralized administrative support, data management capabilities, legal status through which it can execute agreements, and political support that can streamline processes and secure resources.

## PHILOSOPHY OF RESEARCH COLLABORATIONS

The US National Institute of Allergy and Infectious Diseases (NIAID) supports collaborative, government-to-government research partnerships with the Ministries of Health (MoH) of Indonesia, Mali, Guinea, Liberia, Mexico, and the Democratic Republic of the Congo to establish long-term research capacity in preparation for outbreaks like COVID-19. Unlike the traditional investigator-to-investigator model of international collaborative work, where a research partnership is anchored around a specific and limited study or grant, NIAID has developed a “warm base” model of collaboration, where sustained research support independent of a specific study or grant forms a foundation that can be rapidly leveraged for any study. Between outbreaks, these partnerships can focus on infectious disease research priorities of the host nations, such as acute febrile illness in Indonesia [2] and respiratory disease in Mexico [3]. When COVID-19 cases began to surge in January 2020, these warm base partnerships were quickly equipped with diagnostic testing reagents for anticipated COVID-19 studies, which proved valuable when the NIAID partner laboratory in Mali detected the first two COVID-19 cases in that country on March 25, 2020 [4]. As cases continued to rise, normal research activities in each warm base partnership were safely paused so that efforts could focus on COVID-19. In several countries, including Indonesia, the warm base partnership was the only existing structure in place to facilitate the needed rapid, unexpected research.

## RESEARCH PARTNERSHIP IN INDONESIA

Indonesia’s approach to COVID-19 research demonstrates the success of the warm base model and how international research collaborations can be maintained and expanded during a pandemic. Like most countries, Indonesia has been severely affected by COVID-19, with 1 691 658 cases and 46 349 deaths as of May 5th, 2021 [5], though reported numbers likely underestimate the full burden of the disease.

**Figure:**
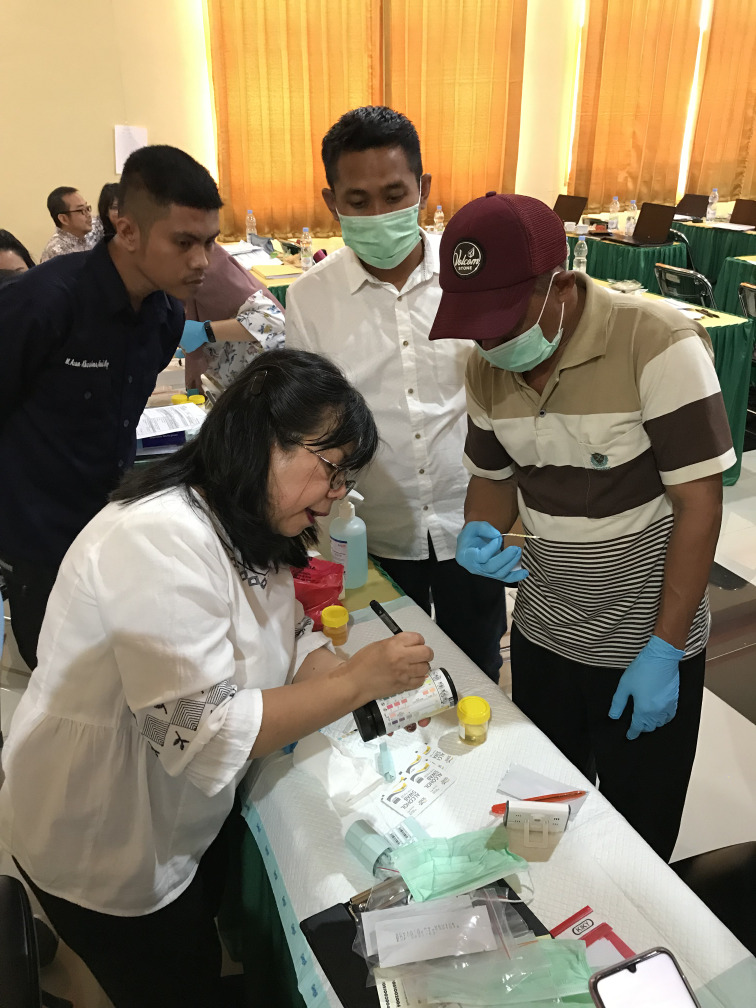
Photo: INA-RESPOND Laboratory Director conducting training at local health centers in Indonesia prior to the COVID-19 pandemic (from Aaron Neal’s own collection, used with permission).

**Figure 1:**
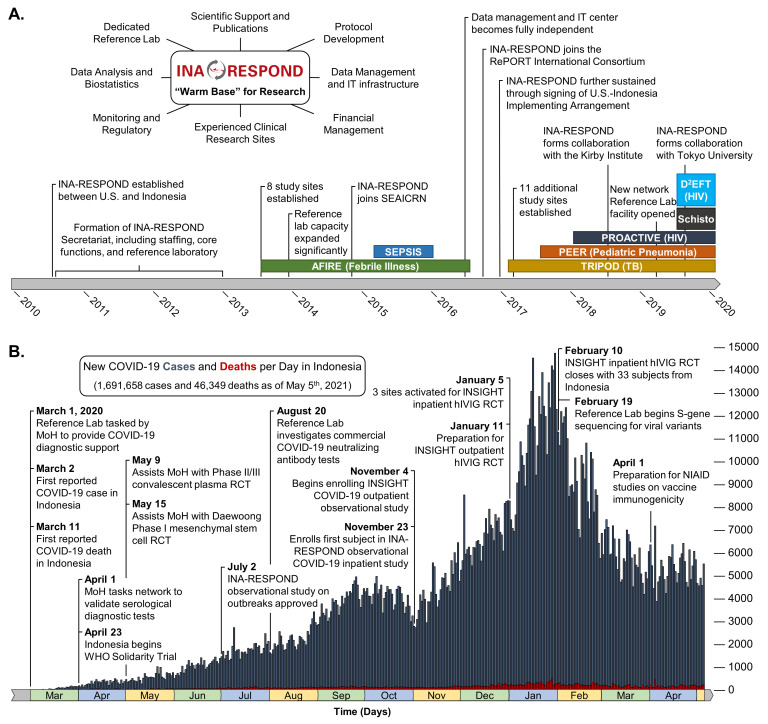
INA-RESPOND warm base model and timelines of network development (**A**), and the COVID-19 pandemic in Indonesia listing network activities (**B**). MoH – Ministry of Healh, RCT – randomized controlled trial; hIVIG – hyperimmue intravenous immunoglobulin.

A significant proportion of Indonesia’s clinical research is conducted by the Indonesia Research Partnership on Infectious Disease (INA-RESPOND) network, a collaborative partnership between NIAID, the Indonesia National Institute of Health Research and Development (NIHRD), and 19 large hospitals and medical facilities across 8 major islands of the archipelago. The network was formed in 2010 in response to a 2007 request from the MoH for greater scientific engagement with the US. Through bilateral support, significant research infrastructure was established over the next 3 years, allowing INA-RESPOND to complete observational studies on sepsis [6] and the etiologies of fever causing hospitalization [2]. The success of these studies led to renewal of the partnership and the initiation of observational cohort studies on HIV (NCT03663920) and tuberculosis (NCT02758236), an observational study on causes of pediatric pneumonia (NCT03366454), an interventional study on HIV treatment (NCT03017872), and a diagnostic study on *Schistosoma japonicum* infection (NCT03870204). As INA-RESPOND has grown, it has also begun collaborating with external groups at the Kirby Institute, Tokyo University, the RePORT consortium, and the INSIGHT network. Today, the network maintains a core secretariat that oversees finance, procurement, study monitoring, data management and analysis, protocol writing, manuscript preparation, and operations oversight, as well as a central reference laboratory that has become one of Indonesia’s most advanced research and diagnostic laboratories (**Figure 1****,** Panel A).

## SHIFTING TO A COVID-19 FOCUS

Once COVID-19 cases began appearing in Indonesia, INA-RESPOND was available and prepared to support the MoH with a rapid response (**Figure 1****,** Panel B). The prior ten years of investment into research infrastructure, scientific capacity, and relationship building meant that the MoH could immediately leverage the network, beginning with the central reference laboratory. Despite a global shortage of key diagnostic test reagents and personal protective equipment, the INA-RESPOND laboratory was sufficiently stocked from ongoing study activities and general warm base procurements. Any SARS-CoV-2-specific reagents and controls that were still needed were obtained and delivered by collaborating US partners. After meeting this immediate public health need, the MoH requested INA-RESPOND to provide additional support with 1) the provision of diagnostic services when requested by health authorities, 2) reporting the first epidemiologic investigation, 3) evaluating the performance of antibody- and antigen-based diagnostic products, 4) implementing the WHO Solidarity Trial on COVID-19 therapeutics [7], and 5) preparing for future vaccine and therapeutic trials. Though these sudden assignments would delay completion of ongoing studies, the network decided to prioritize the critical COVID-19 mitigation endeavours.

Two weeks after identification of the first COVID-19 case in Jakarta, social distancing policies began in Indonesia. During this time, INA-RESPOND modified its standard operations so that secretariat activities were performed remotely, ensuring continuity of important research while also protecting the network’s staff and volunteers. Ongoing HIV and tuberculosis studies were safely placed on temporary hold. Ongoing schistosomiasis and pediatric pneumonia studies were reaching their end and began the close-out process remotely. This pause in INA-RESPOND research allowed the network and investigators to shift focus and resources entirely to COVID-19 research and MoH support. The reference laboratory continued to perform centralized diagnostic testing for the MoH, while 10 of 19 network sites began participating in the WHO Solidarity trial, with the first site activating on April 23, 2020. Additionally, INA-RESPOND implemented an outpatient observational study with INSIGHT (NCT04385251), a general outbreak observational study to include hospitalized COVID-19 patients (NCT04339179), and a randomized double-blind, placebo-controlled trial of hyperimmune intraveneous immunoglobulin with INSIGHT (NCT04546581). These and other COVID-19 studies are undergoing expedited review, drug importation, and implementation using processes instituted for this purpose and facilitated by the extensive experience and government-to-government relationships within the network.

## LESSONS FOR COLLABORATIONS FROM COVID-19

Pivoting the INA-RESPOND research portfolio to support the MoH and maintain relevance during the COVID-19 pandemic has reinforced key research principles [8] and revealed new ways to nurture collaborations. Fundamentally, a partnership like INA-RESPOND cannot be rushed into existence and takes years of building capacity, developing research expertise, and fostering trust to thrive in a pandemic scenario. Efforts to establish warm base research partnerships in preparation for an urgent threat like COVID-19 must be undertaken during times of calm and nurtured through rigorous research of benefit to the host nation and broader international community [9]. Indonesia’s fortunate access to an experienced, well-equipped warm base of in-country scientists through INA-RESPOND enabled the MoH to overcome COVID-19 diagnostic testing shortfalls and engage in the international effort to understand a sudden pandemic.

Though INA-RESPOND was generally prepared for a pandemic scenario, an early realization was that the network lacked an ongoing, open-ended observational protocol to capture clinical and demographic data on early COVID-19 cases. Maintaining a sentinel detection protocol would allow the network to immediately enroll eligible participants and collect critical data to inform public health practice in Indonesia, regardless of the disease or its novelty. By the end of April 2020, network and NIAID staff had collaboratively developed and received ethical approval for a sentinel detection protocol (NCT04339179). Importantly, the expedited development of the protocol did not bypass normal procedures for scientific and ethical evaluation since clinical research conducted during an epidemic must adhere to the same principles as research conducted between epidemics [10]. With an approved protocol and the already established infrastructure and resources of the INA-RESPOND warm base, the network could begin collecting data on COVID-19 and any future epidemic in Indonesia.

As INA-RESPOND has grown, it has pursued increasingly complex clinical research, beginning with simple observational studies and building to interventional studies using therapeutics approved outside of Indonesia. While this gradual development of capacity and experience has been central to the success of the network, INA-RESPOND’s lack of experience with advanced investigational new drug (IND) studies proved to be a barrier to participating in an early interventional trial of remdesivir in severe COVID-19 patients. This understandable outcome reinforces the need to establish and build clinical research networks in times of calm so that they are prepared and experienced enough when emergency situations arise. Equally important is the need to establish the capacity in-country rather than relying on experienced foreign research teams descending upon a country to conduct their own research and leave. The former is not only more equitable and beneficial to the host nation, but in the restricted travel environment of COVID-19, it is the only reliable way to complete the research.

## CONCLUSION

The long-term investment of the Indonesian and US governments in INA-RESPOND as a warm base for clinical research in Indonesia has proven invaluable during the ongoing COVID-19 pandemic. As with COVID-19, the next pandemic remains unpredictable. To maintain capacity to mount a research response to future pandemics, clinical research networks like INA-RESPOND should be nurtured as a global resource and sustained as a successful alternative to the traditional investigator-to-investigator model of international collaborative research.
